# Novel X-Linked Genes Revealed by Quantitative Polymerase Chain Reaction in the Green Anole, *Anolis carolinensis*

**DOI:** 10.1534/g3.114.014084

**Published:** 2014-08-28

**Authors:** Michail Rovatsos, Marie Altmanová, Martina Johnson Pokorná, Lukáš Kratochvíl

**Affiliations:** * Department of Ecology, Faculty of Science, Charles University in Prague, Prague, 128 44; † Institute of Animal Physiology and Genetics, Academy of Sciences of the Czech Republic, Liběchov, 277 21, Czech Republic

**Keywords:** gene dosage, lizard, qPCR, sex chromosomes, sex determination, genetics of sex

## Abstract

The green anole, *Anolis carolinensis* (ACA), is the model reptile for a vast array of biological disciplines. It was the first nonavian reptile to have its genome fully sequenced. During the genome project, the XX/XY system of sex chromosomes homologous to chicken chromosome 15 (GGA15) was revealed, and 106 X-linked genes were identified. We selected 38 genes located on eight scaffolds in ACA and having orthologs located on GGA15, then tested their linkage to ACA X chromosome by using comparative quantitative fluorescent real-time polymerase chain reaction applied to male and female genomic DNA. All tested genes appeared to be X-specific and not present on the Y chromosome. Assuming that all genes located on these scaffolds should be localized to the ACA X chromosome, we more than doubled the number of known X-linked genes in ACA, from 106 to 250. While demonstrating that the gene content of chromosome X in ACA and GGA15 is largely conserved, we nevertheless showed that numerous interchromosomal rearrangements had occurred since the splitting of the chicken and anole evolutionary lineages. The presence of many ACA X-specific genes localized to distinct contigs indicates that the ACA Y chromosome should be highly degenerated, having lost a large amount of its original gene content during evolution. The identification of novel genes linked to the X chromosome and absent on the Y chromosome in the model lizard species contributes to ongoing research as to the evolution of sex determination in reptiles and provides important information for future comparative and functional genomics.

The green anole, *Anolis carolinensis* (ACA), is a member of the highly diversified genus *Anolis* (family Dactyloidae), which traditionally serves as a model group for a vast spectrum of research, recently including, for example, genetics (Novick *et al.* 2009; Piskurek *et al.* 2009; Alföldi *et al.* 2011; Rovatsos *et al.* 2014a), cytogenetics (Castiglia *et al.* 2013; Gamble *et al.* 2014), physiology (Dalla Valle *et al.* 2010; Wu *et al.* 2010; Johnson *et al.* 2011; Murphy and Thompson 2011), behavior (Johnson and Wade 2010), developmental biology (Koshiba-Takeuchi *et al.* 2009; Eckalbar *et al.* 2012), and evolutionary ecology (Losos *et al.* 1998; Losos 2009; Kolbe *et al.* 2011). The green anole was the first nonavian reptile selected for whole-genome sequencing (Alföldi *et al.* 2011), and draft genomes of other reptiles followed shortly thereafter (Castoe *et al.* 2011; St John *et al.* 2012; Vonk *et al.* 2013; Wang *et al.* 2013). Public access to the annotated genome through the Ensembl (http://www.ensembl.org; Flicek *et al.* 2014) and National Center for Biotechnology Information (http://www.ncbi.nlm.nih.gov) databases have promoted ACA to the status of a key model reptile species, attracted the attention of the global scientific community, and contributed to a recent increase in publications addressing comparative and functional genomics in squamate reptiles (Fujita *et al.* 2011; Miller *et al.* 2012; Bar-Yaacov *et al.* 2013; Eckalbar *et al.* 2013; Ezaz *et al.* 2013; Gautam *et al.* 2013).

The ACA karyotype consists of 2n = 36 chromosomes, with six pairs of macrochromosomes and 12 pairs of microchromosomes (Matthey 1931). Although pioneering studies had indicated that ACA possesses a genotypic sex determination (Viets *et al.* 1994), and because the sex chromosomes are homomorphic, these chromosomes had long remained unidentified. During the whole-genome sequencing, it was proven that ACA possesses the XX/XY system of sex chromosomes. The X chromosome (ACAX) was identified at that time using fluorescent *in situ* hybridization of 11 bacterial artificial chromosomes (BACs) containing loci from two contigs. These BACs hybridized to the p arms of two microchromosomes in females but only to the p arm of a single microchromosome in males (Alföldi *et al.* 2011), thus suggesting that these sequences are specific to the X and absent on the Y chromosome. The Y chromosome has not yet been identified, but it is assumed to be another microchromosome (Alföldi *et al.* 2011). The known X-linked region of ACA includes approximately 106 genes (National Center for Biotechnology Information) with orthologs linked to chromosome 15 of the chicken (*Gallus gallus*, GGA). Alföldi *et al.* (2011) speculated that additional X-linked genes might be present on unanchored scaffolds in the AnoCar 2.0 assembly.

Recent studies have demonstrated that the ACA X-linked region is X-linked not only among anoles (Gamble *et al.* 2014; Rovatsos *et al.* 2014a) but also across most phylogenetic lineages of iguanas (Pleurodonta; Rovatsos *et al.* 2014b). The origin of sex chromosomes in iguanas can in fact be traced back to the basal splitting of this group that occurred during the Cretaceous period, and their age can therefore be comparable with the age of sex chromosomes in birds or viviparous mammals. Taking into account that female heterogamety and environmental sex determination have been reported from dragon lizards and chameleons (Acrodonta), the closest outgroup of iguanas (Ezaz *et al.* 2005; Pokorná and Kratochvíl 2009; Young *et al.* 2013), it seems that these XX/XY sex chromosomes constitute a synapomorphy of iguanas.

In contrast to mammals and birds (Ferguson-Smith and Trifonov 2007; Griffin *et al.* 2007), our knowledge about ancestral karyotypes and rates of chromosomal evolution in the majority of the other lineages of amniotes is still limited. Only recent analyses based on gene mapping, chromosomal painting, and whole-genome sequencing have shown that the slow rate of interchromosomal rearrangements is likely to be characteristic for all sauropsids and not just for birds, which constitute their inner group (Matsuda *et al.* 2005; Srikulnath *et al.* 2009; Alföldi *et al.* 2011; Pokorná *et al.* 2011, 2012; Uno *et al.* 2012; Srikulnath *et al.* 2013; Young *et al.* 2013). All X-linked genes in ACA known before the current study have orthologs on chicken chromosome 15 (GGA15). We therefore can assume that the entire GGA15 could be highly homologous to ACAX. Nevertheless, although sauropsids possess a relatively low rate of interchromosomal rearrangement, it has been shown in birds that their intrachromosomal rearrangements occur rather frequently (Völker *et al.* 2010; Skinner and Griffin 2012; Lithgow *et al.* 2014).

In this study, we identified numerous novel X-linked genes in ACA using quantitative fluorescent real-time polymerase chain reaction (qPCR) to compare relative gene doses between male and female genomic DNA (Nguyen *et al.* 2013; Gamble *et al.* 2014; Gamble and Zarkower 2014; Rovatsos *et al.* 2014a,b). The putative X-linked genes were selected from eight unanchored scaffolds sharing homology to GGA15, as predicted by the Genomicus database (http://www.genomicus.biologie.ens.fr/genomicus-75.02/cgi-bin/search.pl; Louis *et al.* 2013). We tested whether all these scaffolds are X-linked in ACA and how frequent were intrachromosomal rearrangements of this chromosome during the independent evolutionary histories of chicken and green anole.

## Materials and Methods

Total genomic DNA was isolated using the DNeasy Blood & Tissue kit (QIAGEN) from the blood of a male and a female of ACA. Primer pairs were designed on Primer3 software (Ye *et al.* 2012) according to ACA sequences from the GenBank database (http://www.ncbi.nlm.nih.gov/genbank; Benson *et al.* 2013) for amplifying putative X-linked loci on scaffolds with homology to GGA15 and the known X-linked regions of ACA (Alföldi *et al.* 2011). Additional primer pairs were designed for autosomal genes localized to ACA chromosome 6, chromosome 5, and for the single-copy gene EF1a, which was used for normalization of the gene dosages in the qPCR analyses (Table 1 and Supporting Information, Table S1).

**Table 1:** Relative gene dosage ratios between sexes of *Anolis carolinensis* revealed by quantitative polymerase chain reaction

Gene Symbol	GenBank Gene ID	Topology in *Anolis carolinensis*	Relative gene dosage Between Sexes
*EF1a*	100566578	NW_003338888.1	−
*FBXW7*	100554110	Chromosome 5; NC_014780.1	1.08
*NHP2L1*	100552266	Chromosome 5; NC_014780.1	1.08
*ADARB2*	100560912	Chromosome 6; NC_014781.1	1.03
*ACAD10*	100557969		0.53
*CMKLR1*	100559738		0.48
*SNAP29*	100554635	NW_003338829.1	0.38
*SDF2L1*	100562697		0.47
*HIRA*	100556463		0.62
*SEPT5*	100563096		0.48
*MLEC*	100557313		0.31
*TBC1D10A*	100558296		0.38
*CIT*	100553665		0.44
*DTX1*	100559683	NW_003338885.1	0.53
*DDX54*	100560281		0.49
*IQCD*	100560479		0.51
*PLBD2*	100561071		0.51
*LHX5*	100555029		0.64
*PUS1*	100562702		0.48
*EP400*	100562901		0.52
*FBRSL1*	100563489		0.52
*GOLGA3*	100563881	NW_003338911.1	0.46
*ZDHHC8*	100561780		0.48
*TRMT2A*	100564671		0.53
*DGCR8*	100561976		0.48
*GAS2L1*	100567612		0.32
*SMTN*	100553012	NW_003338964.1	0.33
*SLC7A4*	100554971		0.47
*B3GNT4*	100566391		0.45
*CLIP1*	100566771	NW_003338970.1	0.63
*KNTC1*	100567355		0.52
*KDM2B*	100557128		0.61
*ORAI1*	100556544		0.53
*WDR66*	100557915	NW_003339097.1	0.50
*LRRC43*	100558501		0.54
*MLXIP*	100558305		0.54
*FICD*	100557652		0.38
*SART3*	100557456	NW_003339461.1	0.53
*TMEM119*	100557060		0.54
*BCR*	100554393		0.44
*SPECC1L*	100554592	NW_003339495.1	0.32
*ADORA2A*	100554785		0.37

Gene names and chromosomal position data follow GenBank database (http://www.ncbi.nlm.nih.gov/genbank).

The qPCR was carried out in a LightCycler II 480 (Roche Diagnostics). All samples were run in triplicates. A 15-μL reaction was performed, containing 2 ng of genomic DNA, 7.5 μL of SYBR Premix Ex Taq II (Takara), and 0.3 mM of each primer. The cycling conditions were 95° for 3 min, followed by 44 amplification cycles of 95° for 15 sec, 56° for 30 sec, 72° for 30 sec, and ending with a melting curve analysis to monitor for potential nonspecific products. The quantification cycle values (crossing point, Cp) were calculated with LightCycler 480 software (v. 1.5.0) according to the second derivative maximum algorithm.

The gene dosage of each studied gene was calculated from Cp values and subsequently normalized to the gene dosage of the single copy gene EF1a based on the equation: R = [2^Cp gene^/2^Cp EF1a^]^−1^ (Cawthon 2002). Finally, the relative gene dosage ratio (r) between sexes was calculated for each gene as r = R_male_/R_female_. Since ACA has the XX/XY sex determination system, we expected a relative gene dosage ratio (r) of 0.5 for X-linked genes missing on the Y chromosome and 1.0 for autosomal or pseudoautosomal genes.

## Results

**Figure 1:**
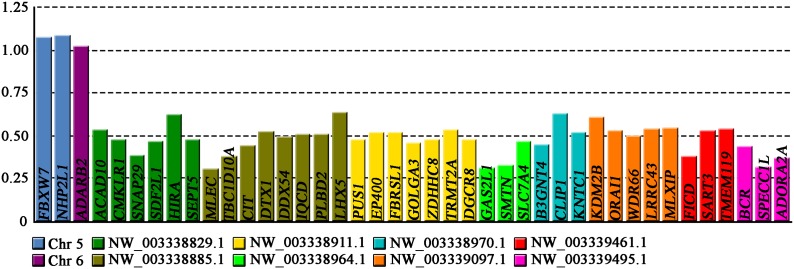
Male-to-female relative gene dosage ratios for genes tested by quantitative polymerase chain reaction in *A. carolinensis*. Value 1.0 is expected for autosomal or pseudoautosomal genes, while value 0.5 is consistent with X-specific position.

The relative gene dosage ratios between males and females were estimated by qPCR for two genes from chromosome 5 (contig No. NC_014780), one gene from chromosome 6 (NC_014781), six genes from the known X-linked region of ACA (NW_003338829), and 32 putative X-linked genes assigned to seven unanchored scaffolds (see Figure 1, Table 1, Table 2, and Table S1). In all cases, the values from the gene EF1a were used for normalization.

**Table 2:** List of contigs from the ACA genome project shown to be X-linked

ACA X-Linked Contigs	Contig Size, bp	Number of Genes	Studied Genes by qPCR	Percentage of Studied Genes per Contig
NC_014783	3 271 537	58	5*^a^*^,^*^b^*	9
NW_003338829	1 779 868	48	9*^a^*^,^*^b^*^,^*^c^*	19
NW_003338885	1 258 094	45	8	18
NW_003338911	1 083 274	17	7	41
NW_003338964	831 895	37	4*^a^*^,^*^b^*	11
NW_003338970	834 740	19	6*^a^*^,^*^b^*	32
NW_003339097	526 944	11	5	45
NW_003339461	147 151	5	3	60
NW_003339495	117 443	10	3	30

Presented are the contig size, gene content, and number of genes tested by qPCR per contig. ACA, *Anolis carolinensis*; qPCR, quantitative polymerase chain reaction.

a Data from Rovatsos *et al.* 2004a.

b Data from Rovatsos *et al.* 2014b.

c Data from Gamble *et al.* 2014.

Our qPCR results confirmed the autosomal position of the genes located in chromosomes 5 and 6 of ACA. As expected for autosomal or pseudoautosomal genes, their relative gene dosage ratios were very close to, and did not differ significantly from, the expected value of 1.0 (*t*-test, *P* = 0.08). In addition, the six genes from the known X-linked region of ACA had male to female gene dosage ratios ranging from 0.38 to 0.62 (mean 0.50), which did not differ significantly from the expected value for X-linkage of 0.5 (*t*-test, *P* = 0.90). The 32 putative X-linked genes demonstrated ratios varying from 0.31 to 0.64 (mean 0.48), which also did not vary significantly from the expected 0.5 (*t*-test, *P* = 0.16). Thus, this leads to the conclusion that these genes are localized to the X chromosome of ACA and are absent from Y. We detected no autosomal or pseudoautosomal genes.

**Figure 2:**
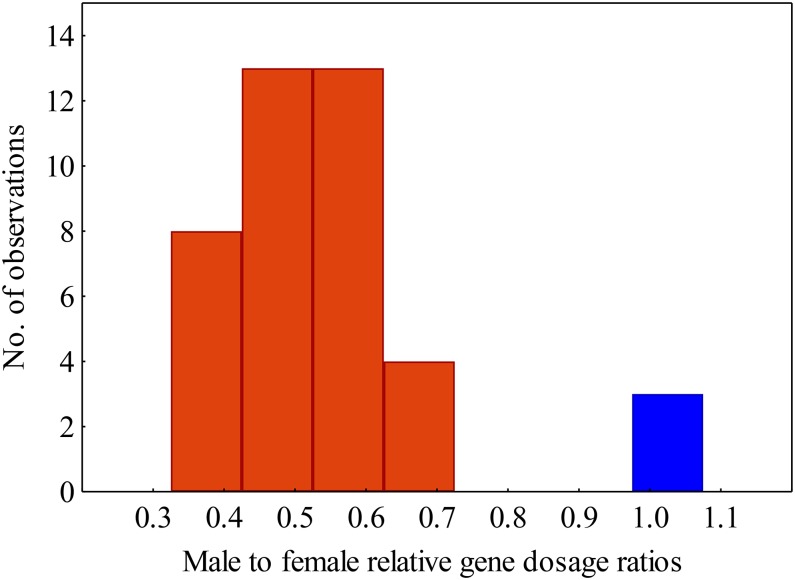
Distribution of male-to-female relative gene dosage ratios in the autosomal (blue) and X-specific (red) genes.

Our qPCR approach identified X-specific genes robustly, since the relative gene dosage ratios for the genes located on autosomes (chromosomes 5 and 6) differ significantly from those of the X-linked genes (analysis of variance, *P* = 0.004). The two sets are thus clearly distinguishable, without overlap or intermediate values (Figure 2).

## Discussion

The first X-linked genes in ACA had been discovered during the whole-genome sequencing project (Alföldi *et al.* 2011) by *in situ* hybridization of 11 BACs containing DNA fragments from two contigs (NC_014783 and NW_003338829). Those two contigs include 58 and 48 genes, respectively. Subsequent studies had tested five genes (*TMEM132D*, *CCDC92*, *ATP2A2*, *PEBP1*, and *MED13L*) from the NC_014783 and three genes (*PPM1F*, *PI4KA*, and *CLTCL1*) from the NW_003338829 contig (Gamble *et al.* 2014; Rovatsos *et al.* 2014a,b) by qPCR for relative gene dosage between sexes, thereby verifying their X-linkage in ACA. Four more X-specific genes (*GAL3ST1*, *ZCCHC8*, *CUX2*, and *SH2B3*) from the scaffolds NW_003338964 and NW_003338970 were discovered by qPCR (Rovatsos *et al.* 2014b). These genes were chosen because they have orthologs linked to GGA15 as the previously known ACAX genes. This work provided the first direct evidence that additional X-linked genes exist on unanchored scaffolds. In the present study, we verified that these two unanchored scaffolds indeed contain X-specific genes by testing six additional genes located within them using qPCR (Table 1). Furthermore, we demonstrate that an additional 26 genes located on five unanchored scaffolds are also part of the ACA X chromosome (Table 1). Using qPCR, we tested 11–60% of the gene content per scaffold (Table 2). Assuming that all genes included on those scaffolds are localized to the ACA X chromosome, we can increase the number of known ACA X-linked genes from 106 (Alföldi *et al.* 2011; Gamble *et al.* 2014; Gamble and Zarkower 2014; Rovatsos *et al.* 2014a,b) to 250. According to the Galgal 4.0 assembly, the GGA15 chromosome has a size of 12.66 Mbp with approximately 430 genes, which suggests that ACAX may contain more genes than the 250 identified here.

Despite intensive study of the ACA sex chromosomes (Alföldi *et al.* 2011; Gamble *et al.* 2014; Rovatsos *et al.* 2014a,b) only limited data about the gene content of the Y chromosome is available. Gamble and Zarkower (2014) identified a partial sequence from the Y-specific gene *RTDR1Y* using restriction site-associated DNA sequencing. In addition, the identification of many ACA X-specific genes localized to distinct contigs (Table 1 and Table 2) indicates that the ACA Y-chromosome is highly degenerated and that it had lost much of its original content during evolution.

In the current study, we detected no gene with an ortholog at GGA15 having a relative gene dosage ratio between male and female consistent with an autosomal or pseudoautosomal position. Using a similar qPCR approach, Gamble and Zarkower (2014) had tested two ACA genes (*RTDR1* and *GNA2*) located in a short contig composed from only three genes for relative gene dosage between sexes. *RTDR1* displayed relative gene dosage ratios between male and female consistent with an X-specific position. *GNA2* yielded equal ratios between sexes, thus suggesting that this gene has a gametolog on the Y chromosome (Gamble and Zarkower 2014). Data from the Genomicus database (http://www.genomicus.biologie.ens.fr/genomicus-75.02/cgi-bin/search.pl; Louis *et al.* 2013) indicate, however, that some genes from the GGA15 chromosome are localized to autosomes in ACA (*e.g.*, *ACACB* on ACA chromosome 1 or *SFI1* on ACA chromosome 3). This probably is due to translocations that occurred during the 250 million years of divergence between ACA and GGA. The test for putative pseudoautosomal position of *GNA2* requires further experimental work.

All studied genes were X-specific and not present on the Y chromosome in ACA. We did not detect any evidence for autosomal or pseudoautosomal position of the genes with orthologs linked to GGA15. It is therefore possible that the pseudoautosomal region in ACA is small or absent. Alternatively, a pseudoautosomal region in ACA could be homologous to a chromosome other than GGA15 and, as such, it could not be determined using our approach. These hypotheses should be tested by, for instance, observing the behavior of sex chromosomes during male meiosis.

**Figure 3:**
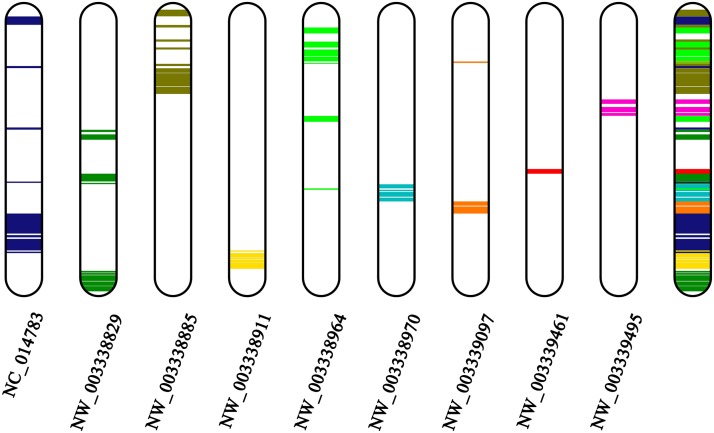
Homology between nine *A. carolinensis* contigs and the *Gallus gallus* chromosome 15. The homology was predicted by the Genomicus database (http://www.genomicus.biologie.ens.fr/genomicus-75.02/cgi-bin/search.pl).

Comparison of the topology between the X-linked ACA contigs and GGA 15 (Figure 3), as illustrated by the Genomicus database (http://www.genomicus.biologie.ens.fr/genomicus-75.02/cgi-bin/search.pl; Louis *et al.* 2013), shows that despite high conservation of the chromosome, several intrachromosomal rearrangements, such as inversions, probably occurred at this chromosome after the divergence of ACA and GGA from their common ancestor. Similarly, no interchromosomal but numerous intrachromosomal rearrangements have been documented in the microchromosomes of chicken, turkey, and zebra finch (Lithgow *et al.* 2014). Several pericentromeric inversions have been revealed in the chromosomal pairs 1–4 of ACA by *in situ* hybridization with BACs, but their functional importance remains unclear (Alföldi *et al.* 2011). We should keep in mind, however, a recent counterexample based on fluorescent *in situ* hybridization mapping of 11 markers whereby a high level of synteny was revealed between ACA chromosome 6 and its homologous chromosome in snakes (Z in colubroid snakes) without any detectable large-scale chromosomal rearrangements (Vicoso *et al.* 2013).

Reptiles (excluding their inner avian group) usually are considered a group with rapid turnover of sex-determining mechanisms (Sarre *et al.* 2004; Organ and Janes 2008). In general, they do indeed exhibit large variability in sex-determining systems (Valenzuela and Lance 2004; Pokorná and Kratochvíl 2009; Gamble 2010). It has been suggested that poikilotherms possess more frequent turnovers of sex chromosomes than do homoiotherms, whose effective thermoregulation can prevent the emergence of sex reversals induced by environmental temperature. Despite their species richness, wide distribution, and enormous ecological and morphologic variability, however, iguanas possess great stability of sex chromosomes (Rovatsos *et al*. 2014b) that is comparable with the well-documented cases of birds (ca 120 Mya according to Mank and Ellegren 2007) and therian mammals (ca 166 Mya based on Veyrunes *et al.* 2008). Although such stability of sex chromosomes has been reported for several poikilothermic amniotes, such as within the turtle family Trionychidae (ca. 95 Mya) (Badenhorst *et al.* 2013) or within colubroid snakes (Matsubara *et al.* 2006; Vicoso *et al.* 2013), where the split between the studied families Viperidae and Colubridae occurred, ca. 36 Mya according to Wiens *et al.* (2006), but even up to 75 Mya according to Wüster *et al.* (2008), to the best of our knowledge, iguanian lizards possess the oldest sex chromosomes currently known among amniotic poikilothermic vertebrates. By reporting the large list of X-specific genes in ACA, our recent contribution enables future comparative study on the evolution of sex chromosomes in iguanas. For example, applying the qPCR approach within lineages derived from basal splitting of iguanas may reveal whether highly differentiated X and Y chromosomes described in ACA evolved via a stepwise series of suppressions of recombination along the iguana Y chromosome, *i.e.*, whether some “evolutionary strata” found in mammals and birds (Lahn and Page 1999; Handley *et al.* 2004; Nam and Ellegren 2008) could be observed also in iguanas.

## Supplementary Material

Supporting Information
