# Supplementary material for: Novel X-Linked Genes Revealed by Quantitative Polymerase Chain Reaction in the Green Anole, Anolis carolinensis
Source: G3 (Bethesda). 2014 Aug 28;4(11):2107–13. doi: 10.1534/g3.114.014084 (PMC4232536; doi:10.1534/g3.114.014084)
Supplement: Supporting Information [file supp_4_11_2107__index.html]

Novel X-Linked Genes Revealed by Quantitative Polymerase Chain Reaction in the Green Anole, Anolis carolinensis — Supporting Information 

# Novel X-Linked Genes Revealed by Quantitative Polymerase Chain Reaction in the Green Anole, *Anolis carolinensis*

## Supporting Information for Rovatsos *et al.*, 2014

**Files in this Data Supplement:**

- Table S1 - Topology of all studied genes in *Anolis carolinensis* (ACA) and in *Gallus gallus* (GGA). Primer pairs were designed for estimating the relative gene dosage using qPCR. Amplicon size was predicted by *in silico* PCR in UCSC Genome Browser (http://genome.ucsc.edu). (.xls, 41 KB)
